# Phototoxic Action Spectrum on a Retinal Pigment Epithelium Model of Age-Related Macular Degeneration Exposed to Sunlight Normalized Conditions

**DOI:** 10.1371/journal.pone.0071398

**Published:** 2013-08-23

**Authors:** Emilie Arnault, Coralie Barrau, Céline Nanteau, Pauline Gondouin, Karine Bigot, Françoise Viénot, Emmanuel Gutman, Valérie Fontaine, Thierry Villette, Denis Cohen-Tannoudji, José-Alain Sahel, Serge Picaud

**Affiliations:** 1 Institut de la Vision, UPMC Univ Paris 06, UMR_S 968, Paris, France; 2 INSERM, U968, Paris, France; 3 CNRS, UMR_7210, Paris, France; 4 Essilor International, Charenton-le-Pont, France; 5 Muséum National d'Histoire Naturelle, Paris, France; 6 Centre Hospitalier National d'Ophtalmologie des Quinze-Vingts, INSERM-DHOS CIC 503, Paris, France; 7 Institute of Ophthalmology, University College of London, London, United Kingdom; 8 Fondation Ophtalmologique Adolphe de Rothschild, Paris, France; 9 Académie des Sciences-Institut de France, Paris, France; University of Florida, United State of America

## Abstract

Among the identified risk factors of age-related macular degeneration, sunlight is known to induce cumulative damage to the retina. A photosensitive derivative of the visual pigment, *N*-retinylidene-*N*-retinylethanolamine (A2E), may be involved in this phototoxicity. The high energy visible light between 380 nm and 500 nm (blue light) is incriminated. Our aim was to define the most toxic wavelengths in the blue-green range on an *in vitro* model of the disease. Primary cultures of porcine retinal pigment epithelium cells were incubated for 6 hours with different A2E concentrations and exposed for 18 hours to 10 nm illumination bands centered from 380 to 520 nm in 10 nm increments. Light irradiances were normalized with respect to the natural sunlight reaching the retina. Six hours after light exposure, cell viability, necrosis and apoptosis were assessed using the Apotox-Glo Triplex™ assay. Retinal pigment epithelium cells incubated with A2E displayed fluorescent bodies within the cytoplasm. Their absorption and emission spectra were similar to those of A2E. Exposure to 10 nm illumination bands induced a loss in cell viability with a dose dependence upon A2E concentrations. Irrespective of A2E concentration, the loss of cell viability was maximal for wavelengths from 415 to 455 nm. Cell viability decrease was correlated to an increase in cell apoptosis indicated by caspase-3/7 activities in the same spectral range. No light-elicited necrosis was measured as compared to control cells maintained in darkness. Our results defined the precise spectrum of light retinal toxicity in physiological irradiance conditions on an *in vitro* model of age-related macular degeneration. Surprisingly, a narrow bandwidth in blue light generated the greatest phototoxic risk to retinal pigment epithelium cells. This phototoxic spectrum may be advantageously valued in designing selective photoprotection ophthalmic filters, without disrupting essential visual and non-visual functions of the eye.

## Introduction

Age-related macular degeneration (ARMD) is one of the major causes of blindness in industrialized countries and it is estimated to be responsible of 22.9% of the cases of blindness and 54.4% of visual impairments in the white American population [1]. Nowadays, 9.1 million Americans over the age of 50 are likely to suffer from an early ARMD [2]. This number is expected to double by 2050 to reach 17.8 million [3]. The severe visual loss due to ARMD is affecting at least 12% of the U.S. and European populations over the age of 80 [2], [4], [5].

Age, smoking, skin color, genetic factors and nutritional antioxidant deficiencies have been identified as risk factors in ARMD [6]. Although implication of light remains controversial, several studies indicate light exposure as a factor in the pathogenesis of ARMD [7], [8], [9], [10], [11], [12], [13]. For instance, the EUREYE study found significant association between blue light exposure and neovascular ARMD in individuals having the lowest antioxidant level [11]. Another study performed on 838 watermen of the Chesapeake Bay showed that patients with advanced ARMD had significantly higher exposure to blue or visible light over the preceding 20 years [8]. Finally, a recent analysis of the epidemiological literature concerning the association between ARMD and sunlight exposure concluded that individuals with more sunlight exposure are at a significantly increased risk of ARMD [14].

Early stages in ARMD are usually associated with the formation of characteristic deposits underneath the retinal pigment epithelium (RPE) and Bruch's membrane, called drusen [15], and with the accumulation of lipofuscin in RPE cells. Lipofuscin accumulates with age in lysozomes as a by-product of the visual cycle and of the incompletely degradation of phagocytosed oxidized photoreceptor outer segments [16], [17], [18]. The major chromophore of lipofuscin is A2E (*N*-retinylidene-*N*-retinylethanolamine), a photosensitizer generating photodynamic damage [19], [20]. The specific toxicity of short wavelengths on the retina is thought to be related to the presence of this intracellular molecule [21].

Studies performed on animal and cellular models were able to demonstrate the toxicity of light and more specifically of the blue spectral range on the RPE and photoreceptor cells. For instance, *in vivo* experiments revealed that photochemical damages exhibit lower dose thresholds in the UVs and in the blue range than for green or red light on the retina [22] of monkey [23], [24], rat [25], [26], [27] and rabbit [28], [29], [30], [31], [32]. These light damages were then modeled on primary or immortalized RPE cells loaded with either oxidized photoreceptor outer segments [33], purified lipofuscin [34], or synthesized A2E [35], [36], [37], [38], [39]. A greater toxicity of blue light was confirmed by exposing human RPE cells loaded with lipofuscin during 48 hours to blue-green light (390–550 nm, 2.8 mW/cm^2^) compared to yellow-red light (550–800 nm, 2.8 mW/cm^2^) [34]. Similarly, exposure to blue light (480±20 nm, 75 mW/mm^2^) induced more cell death on immortalized RPE cells loaded with A2E (ARPE-19 cell line) than green light (545±15 nm, 200 mW/mm^2^) [36]. Blue light-induced cell death was mediated by apoptotic processes involving caspase-3 and p-53 activation [40], [41]. However, in these studies, the light irradiance was not normalized to physiological conditions, meaning (i) no calibration on sunlight spectrum and (ii) no consideration for the eye media filtering. Furthermore, no test was achieved to precisely define the most toxic wavelengths within the entire blue range.

In the present study, our aim was first to calculate sunlight irradiances reaching the retina and second to assess on A2E-loaded RPE cells the light toxicity of 10 nm illumination bands at irradiances normalized to the calculated retinal sunlight irradiances. We have thus defined the most toxic wavelengths in the blue range, which could be precisely removed to best preserve color vision and non-visual functions.

## Results

### Light exposure

**Figure 1:**
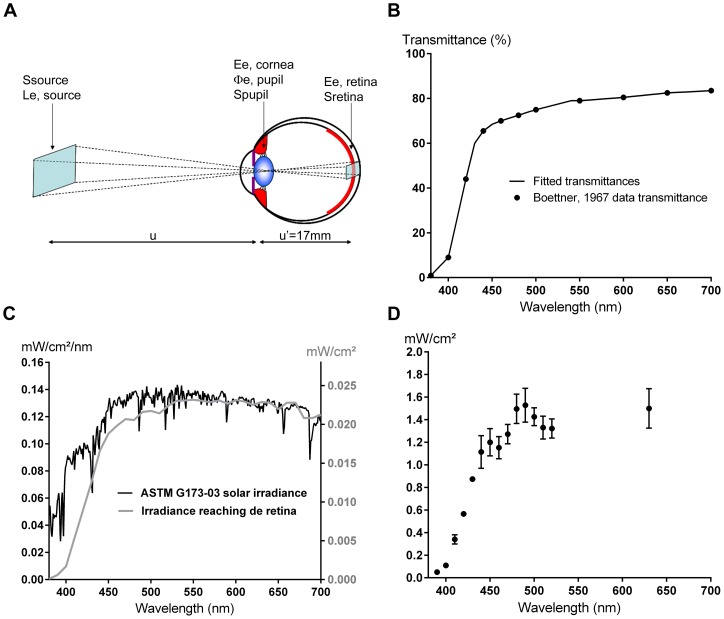
Sunlight irradiance reaching the retina. **A.** Eye/light source model adapted from [61]. The light source is described by its energetic radiance *L*_e,λ,source_(λ) (W/sr/m^2^) measured in the pupil direction and its emitting surface *S*_source_ (m^2^). The source is assumed to be small compared to the distance u (m) between the source and the cornea. The cornea plane, the pupil plane and the nodal planes are assumed to be superimposed. The retina surface *S*_retina_ illuminated by the light source is proportional to the surface of the source *S*_source_. **B.** Percent transmittance reaching the retina fitted from [61]. **C.** ASTM G173-03 solar spectral irradiance (black curve, left axis) and sunlight irradiances reaching the retina (grey curve, right axis). The energetic irradiances reaching the retina were calculated by applying the ocular media filtering onto the referenced solar spectrum. **D.** Irradiances at well-plate level. The light exposure conditions were obtained by applying a multiplying coefficient to the calculated retinal irradiances. Values are expressed as mean ± s.e.m (n = 4 to 6).

To model sunlight exposure of the retina, irradiances for each 10 nm light source were calibrated according to a normalized spectrum obtained by applying natural ocular filters (Fig. 1A and 1B) onto a standard solar spectrum (ASTM G173-03) (see materials and methods, Fig. 1C). The radiometric calculations were based on two main parameters: (i) the energetic radiance of the light source and (ii) the ocular media transmittance adapted from [42] and in accordance with the recent reference transmittance data from CIE 203:2012. The measured irradiances at plate level are shown in Fig. 1D.

**Figure 2:**
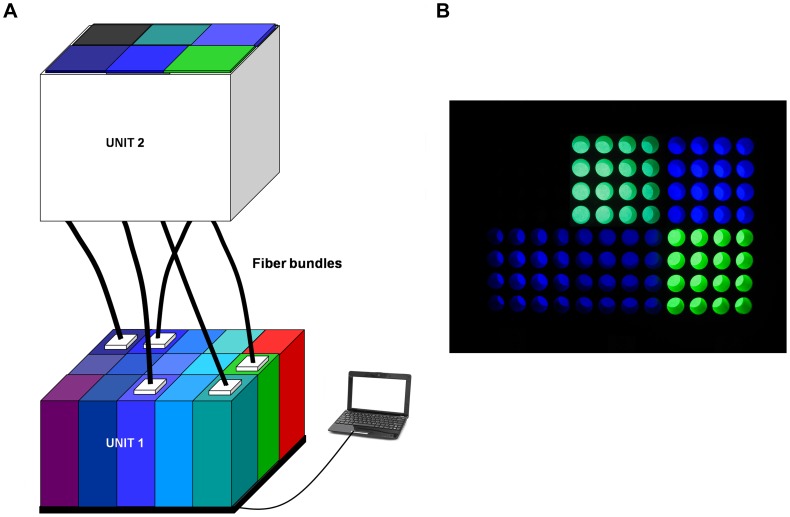
Light emitting device. **A.** The LED-based fibered illumination device is composed of two optical units and of a software control. Unit 1 generates fifteen 10 nm bandwidth illumination channels. Fourteen bands are equally distributed within the blue-green range in 10 nm increments with the first band centered at 390 nm and going up to 520 nm. An additional band is set at 630 nm in the red spectral range. The energetic irradiance delivered by each illumination channel is monitored by computer. Unit 2 is located in the cell incubator and ensures the light uniformity on cells. The two optical units are linked by five independent systems of fiber bundles providing light to five 16-well subdivisions of the 96-well plate. **B.** Representative 96-well plate illumination. Five 16-well subdivisions are simultaneously illuminated with distinct illumination bands while a 16-well subdivision is maintained in darkness.

A LED-based fibered illumination device with computer monitoring was especially designed to apply the normalized light conditions (Fig. 2A and B). The light generator was composed of 14 narrow illumination bands equally distributed within the blue-green range in 10 nm increments with the first band centered at 390 nm and going up to 520 nm. An additional band was set at 630 nm.

### Characterization of A2E-loaded RPE cells

**Figure 3:**
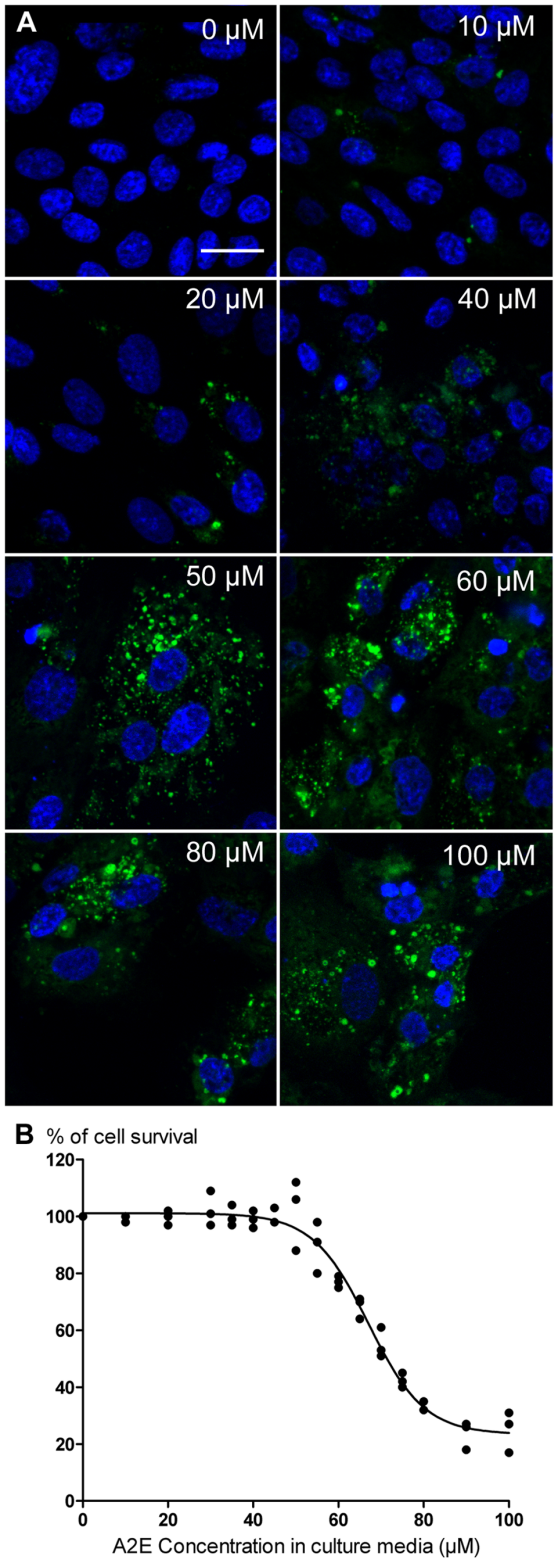
Accumulation and toxicity of A2E in RPE cells. **A**. Confocal imaging of A2E accumulation in RPE cells at various A2E concentrations in the culture medium. Individual cells can be detected by their cell nuclei (blue DAPI staining) while A2E incubation is associated with the apparition of autofluorescent dots (green) in RPE cells. Note the gradual increase in A2E autofluorescence in RPE cells with the increase in A2E concentrations applied in the incubation medium for 6 hours. **B**. Dose response curve of A2E toxicity on RPE cells. Cell survival was quantified with the CellTiter-Glo® assay 24 hours after the 6 hour A2E incubation. A loss of cell survival was detected at A2E concentrations over 45 µM with an IC50 at 67.5 µM. (n = 3, r^2^ = 0.9727). Scale bar represents 20 µm.

**Figure 4:**
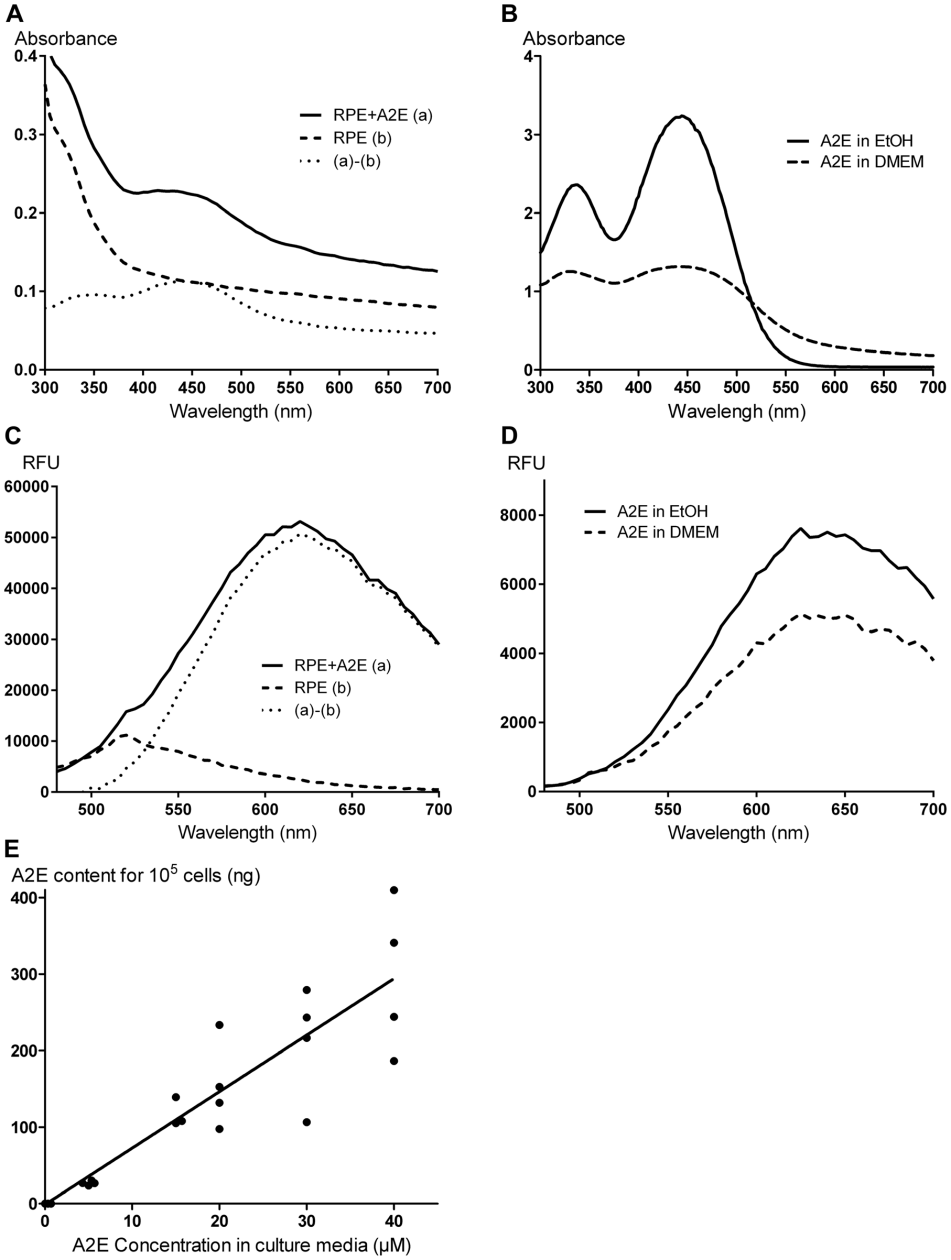
Characterization of the autofluorescence in A2E-loaded RPE cells. **A.** Absorbance of RPE cells treated with 40 µM A2E (A2E+RPE (a), solid line) or A2E-untreated (RPE (b), dashed line). The curve ((a)–(b), dot line) representing the difference of absorption spectra between A2E-loaded RPE cells (a) and A2E-untreated RPE cells (b), shows absorption peaks at 335 nm and 440 nm. **B.** Absorbance spectra of free A2E in pure ethanol (solid line) or in modified DMEM (dashed line). Spectra are similar in both media and A2E displays maxima of absorbance at 335 nm and 440 nm. **C.** Emission spectra of RPE cells treated with 40 µM of A2E (A2E+RPE (a), solid line) or untreated (RPE (b), dashed line) under a 440 nm excitation. The curve ((a)–(b), dot line) representing the difference between the emission spectra in A2E-loaded RPE cells (a) and A2E-untreated RPE cells (b) shows a peak at 620 nm. **D.** Emission spectra of free A2E in pure ethanol (solid line) or in modified DMEM (dashed line) with a 440 nm excitation. Spectra are similar in both media and A2E displays a maximum of emission at 640 nm. **E.** A2E contents quantified by UPLC in RPE cells after 6 hours of incubation in various A2E concentrations (0, 5, 15, 20, 30 and 40 µM) in culture medium. The A2E content in RPE cells increases in a linear way according to the incubated A2E concentration. (n = 4, r^2^ = 0.9955). (RFU: Relative fluorescence unit).

To clarify whether porcine RPE cells can be used as a cellular model of RPE ageing, we first examine their ability to accumulate A2E during A2E incubation. A2E ingestion by RPE cells was suggested by the onset of numerous fluorescent dots visible by microscopic observation in the cytoplasm (Fig. 3A). The increase in fluorescent vesicles within RPE cells was apparently dose-dependent upon the extracellular A2E concentrations (Fig. 3B). To investigate whether these autofluorescent bodies revealed the presence of intracellular A2E, the absorption spectrum of A2E-incubated RPE cells was performed. The A2E incubation at 40 µM generated an absorption peak at 440 nm (Fig. 4A), which was not present in untreated RPE cells. Subtraction of spectra from A2E-loaded cells and untreated cells gave two absorption peaks at 335 and 440 nm, as observed with free A2E dissolved in modified DMEM or in pure ethanol (Fig. 4B). These observations were consistent with previously reported absorption spectra for free A2E [37]. After light excitation at 440 nm, A2E-loaded cells exhibited an emission spectrum peaking at 620 nm (Fig. 4C), close to the A2E emission maximum at 640 nm (Fig. 4D) in DMEM or in pure ethanol. To further confirm the internalization of A2E by RPE cells, A2E was purified from cultured RPE cells and quantified by UPLC allowed A2E detection down to 10 pg (Fig. 4E). In our conditions, the A2E concentration in RPE cells displayed a linear correlation with the A2E concentrations added to the culture medium (r^2^ = 0.9955, n = 4). Untreated RPE cells exhibited a very low endogenous content of A2E (0.032±0.006 ng/10^5^ cells). This observation confirmed the A2E internalization during the 6 hour incubation period, as previously published [37].

In our hands, high A2E concentrations (i.e. 100 µM) appeared toxic to RPE cells even in darkness. Indeed, the cell density seemed reduced at the end of the A2E incubation (Fig. 3A). To finely define toxic A2E concentrations in darkness, cell viability was quantified with the CellTiter-Glo® viability assay on RPE cells incubated with A2E concentrations ranging from 0 to 100 µM. Viable RPE cell signal decreased for A2E concentrations greater than 45 µM. The dose response curve exhibited a sigmoidal shape with an IC50 at 67.5 µM (Fig. 3B). To avoid this direct A2E toxicity, the determination of the phototoxic action spectrum was subsequently determined with RPE cells incubated with A2E at 12.5, 20 or 40 µM.

### Phototoxic action spectrum on A2E-loaded RPE cells

**Figure 5:**
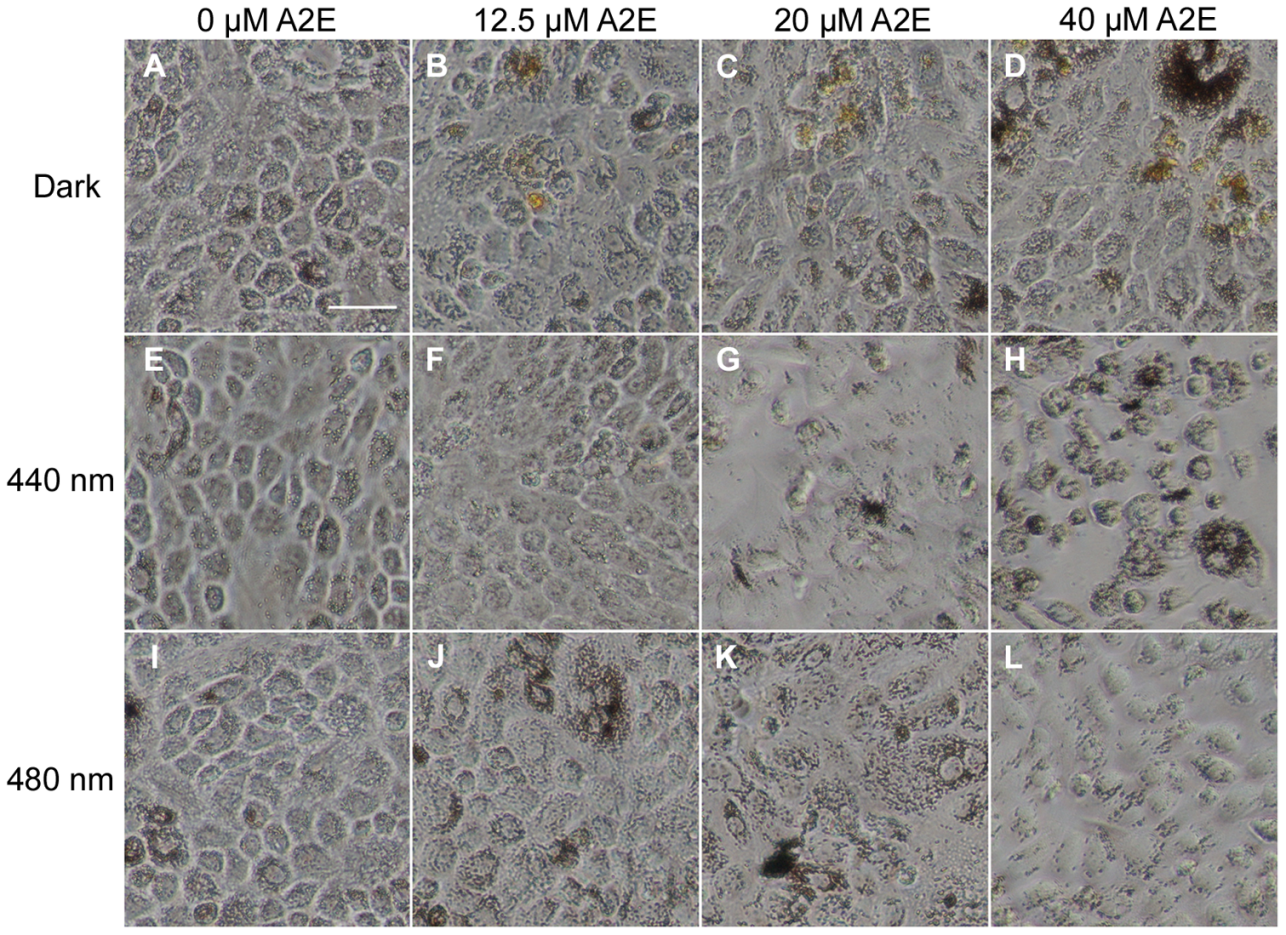
Light-induced morphological changes in A2E-loaded RPE cells. Images of RPE cells were obtained 6 hours after 18 hour light exposure with a 10 nm illumination band centered at 440 nm (**E–H**), at 480 nm (**I–L**) or maintained in darkness (**A–D**). RPE cells were incubated with A2E at 0 µM (**A, E, I**), 12.5 µM (**B, F, J**), 20 µM (**C, G, K**) or 40 µM (**D, H, L**). Note the yellow tint of A2E-loaded RPE cells maintained in darkness (**B–D**). RPE cells treated with A2E at 20 µM (**G**) or 40 µM (H) became round and lost their confluence after their exposure to a 10 nm band centered at 440 nm. By contrast, A2E-loaded RPE cells appeared healthy after their exposure to a 10 nm band centered at 480 nm (**J–L**) similarly as cells maintained in darkness (**B–D**) or A2E-untreated (**A, I**). Scale bar in A represents 20 µm.

To determine the precise action spectrum of light toxicity on RPE cells within the blue-green range, we exposed A2E-loaded cells to fourteen 10 nm illumination bands centered at 390 nm up to 520 nm in 10 nm increments with sunlight normalized irradiances. A2E-loaded RPE cells were exposed to light for 18 hours and examined after a 6-hour rest in darkness. Figure 5 illustrates that the morphology of A2E-loaded RPE cells was affected by light exposure with specific illumination bands. Even with the A2E incubation, cells exposed to the illumination band centered at 480 nm appeared healthy (Fig. 5I–L) as those maintained in darkness (Fig. 5A–D). Morphological changes were evident following the 440 nm light exposure (Fig. 5E–H). These morphological changes were exacerbated on RPE cells incubated with either 20 µM (Fig. 5G) or 40 µM (Fig. 5H) of A2E. Cells tended to round up, to lose their confluence and their density seemed to decrease.

**Figure 6:**
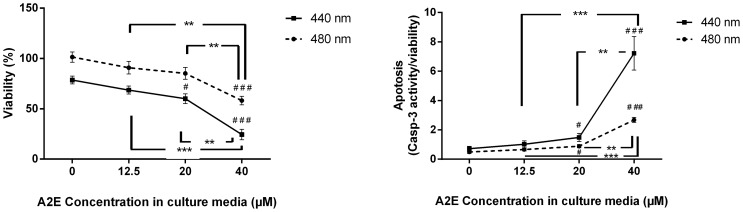
A2E dose response curves of the A2E-elicited phototoxicity on RPE cells. Cell viability (**A**) and cell apoptosis (**B**) were quantified with the ApoTox-Glo™ assay according to the A2E concentrations (0, 12.5, 20 and 40 µM) for RPE cells exposed to the 10 nm illumination bands centered at 440 or 480 nm. Cell viability and apoptosis were normalized with the experimental value obtained for RPE cells maintained in darkness without A2E treatment. The *P*-value was calculated using t-test. For the two illumination bands, statistically significant differences are indicated with respect to the illuminated conditions but in the absence of A2E (^#^p<0.05, ^##^p<0.01, ^###^p<0.001) and when considering two A2E concentrations (*p<0.05, **p<0.01, ***p<0.001).

To quantify the respective cell viability and characterize possible degenerative events caused by light conditions, we used the Apotox-Glo™ assay (Promega) on RPE cells. For each individual well, we measured 1) cell viability, 2) cell apoptosis and 3) cell necrosis. For each A2E concentration and each light condition, the measures were averaged from 4 wells and normalized with respect to the dark control (%). Measurements for each individual light condition were reproduced in at least 4 and up to 6 independent experiments. All data were normalized to the control condition without A2E in the medium and maintained in darkness. This normalization was required to give equivalent weight to all experiments because cell densities slightly varied between experiments and only 5 different light conditions were simultaneously tested in one experiment. The quantification of the RPE cell viability confirmed the phototoxicity of the 440 and 480 nm illumination bands (Fig. 6A) The measurements at both 20 µM and 40 µM of A2E showed statically significant differences with those of cells exposed to light but in the absence of A2E treatment. Similarly, the differences observed between the cell viability at 40 µM of A2E and those at either 12.5 µM or 20 µM of A2E were also statistically significant. These results on the loss of cell viability were consistent with the increase in cell apoptosis for both illumination bands (Fig. 6B). Again, the differences were statistically significant between the measurements at the 20 µM or 40 µM of A2E and those in the absence of A2E as well as between the measurements at 40 µM of A2E and those at either 12.5 µM or a 20 µM of A2E. These results provide evidence for an A2E dose-dependent decrease of RPE cell viability and an A2E dose-dependent increase in cell apoptosis upon both the 440 nm and the 480 nm light exposure.

**Figure 7:**
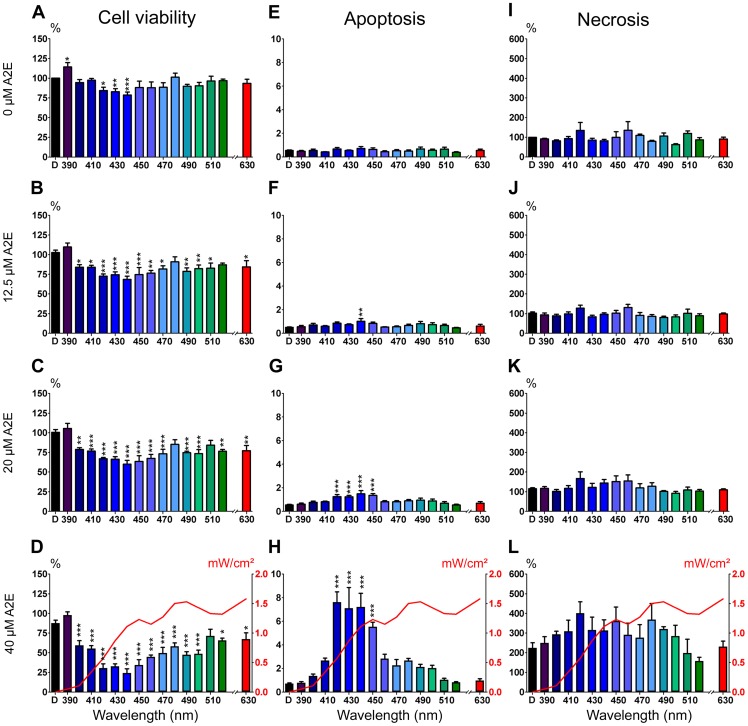
Phototoxic action spectrum on A2E-loaded RPE cells. (**A–D**) Histograms of cell viability according to the 10 nm illumination bands centered from 390 nm to 520 nm and at 630 nm for A2E-untreated RPE cells (0 µM, **A**) or A2E-loaded RPE cells (A2E concentrations:12.5 µM, **B**; 20 µM, **C**; or 40 µM, **D**). Viability levels were normalized with respect to the fluorescent signal measured in dark-maintained A2E-untreated cells (left vertical axis). (**E–H**) Histograms of cell apoptosis according to the 10 nm illumination bands centered from 390 nm to 520 nm and at 630 nm for A2E-untreated RPE cells (0 µM, **E**) and A2E-loaded RPE cells (A2E concentrations: 12.5 µM, **F**; 20 µM, **G**; or 40 µM, **H**). Apoptosis is expressed as the ratio of the caspase-3/7 activity luminescent signal reported to the cell viability fluorescent signal. (**I–L**) Histograms of cell necrosis according to the 10 nm illumination bands centered from 390 nm to 520 nm and at 630 nm for A2E-untreated RPE cells (0 µM, **I**) or A2E-loaded RPE cells (A2E concentrations: 12.5 µM, **J**; 20 µM, **K**; or 40 µM, **L**). Necrosis levels are normalized to the fluorescent signal measured in dark-maintained A2E-untreated RPE cells. Each 10 nm illumination band is designated on the graphs by its central wavelength. The red curves in **D, H** and **L** represent the mean light irradiances (right vertical axis) for each 10 nm illumination band. Statistically significant differences as compared to A2E-untreated cells maintained in darkness (*p<0.05; **p<0.01; ***p<0.001).

When exposures were performed across all 10 nm illumination bands in the absence of A2E treatment, a decrease in cell viability was already detected at 420, 430, 440 nm as compared to cells maintained in darkness (Fig. 7A). With A2E treatment, the decrease in cell viability extended to wavelengths from 400 to 470 nm (Fig. 7B–D). Some viability losses were also observed between 480 and 520 nm and even at 630 nm (Fig. 7B–D). At 12.5 µM, the highest statistically significant differences were observed for the 4 illumination bands centered at 420, 430, 440 and 450 nm. The superimposition of the cell irradiance levels used for each light condition (red curve in Fig. 7D) clearly demonstrated that there is no direct correlation between the irradiance level and the loss in cell viability.

Examination of cell apoptosis by measuring caspase-3/7 activities within living cells showed an increase of the signal with increasing A2E concentrations. Even if no induction of apoptosis was detected in the absence of A2E incubation (Fig. 7E), the increase in caspase-3/7 activities was already statistically significant for the 440 nm illumination band with a 12.5 µM A2E treatment (Fig. 7F). Rising A2E concentration to 20 or 40 µM, cell apoptosis was detected for the 4 illumination narrow bands centered at 420, 430, 440 and 450 nm (Fig. 7G–H). This narrow spectral range inducing programmed cell death was consistent with the spectral bands showing the greater loss of cell viability (Fig. 7A–D). Although a 2-fold increase in cell necrosis was detected by increasing the A2E concentration to 40 µM, the differences were not statistically significant for none of the light condition as compared to cells maintained in darkness (Fig. 7I–L). Therefore, cell necrosis does not appear to contribute to the loss in cell viability defined with a precise light specificity. These results indicate that A2E contributes to the RPE cell photodamage that leads to cell apoptosis with a greater sensitivity in the 415–455 nm spectral range.

## Discussion

Light is often considered as a potential risk factor for RPE dysfunction, and thus ARMD development [11]–[17], [41]. To investigate the phototoxic action spectrum in aging RPE cells, the disease has been modeled by loading cultured RPE cells with A2E [35], [36], [37], [38], [39], a photosensitizer, which accumulates in these cells during aging as in ARMD patients [38]–[42]. Previous studies on this subject have used either an immortalized human RPE cell line, ARPE-19 [37] or human RPE cells [38]. These different cultured RPE cells can accumulate A2E following a mere incubation [37]. The cellular A2E accumulation was indicated by the apparition of fluorescent subcellular structures colocalized with the lysozomal compartment in both ARPE-19 and human RPE cells [37], [38]. A2E incorporation into cultured RPE cells was further demonstrated by cell quantification showing a dose-dependent increase with A2E incubation concentrations. In the present study, we have taken advantage of the easy access to porcine eyes to generate primary porcine RPE cell cultures. By loading cells with A2E, we similarly observed intracellular fluorescent dots with increasing densities according to A2E concentrations. After light excitation at 440 nm, the A2E emission maximum was shifted from 640 to 620 nm between the A2E free form in modified DMEM and the A2E intracellular form. This kind of emission shift has been previously observed from 610 to 565 nm after light excitation at 380 nm [37]. However, we did not investigate the fate of A2E and its transformation into different derivatives upon light, as previously reported [43]. A2E intracellular quantification confirmed its incorporation into porcine RPE cells in a dose-dependent manner, as previously reported in human RPE cells [37]. The resulting A2E contents in our porcine RPE cells were in the same ranges as those reported in cultured A2E-loaded ARPE-19 cells. Moreover, our cells treated with 12.5 µM of A2E displayed similar intracellular content to previously described elderly human donor eyes [37]. These observations indicate that our A2E-loaded porcine RPE cells behave like previously described models, providing thereby an adequate *in vitro* ARMD model.

A2E was found to be a photosensitizer triggering cell death in human RPE cells [38]. The cell viability loss after light exposure was previously shown using the MTT colorimetric assay measuring a metabolic activity [38]. In A2E-loaded ARPE-19 cells, light-induced degeneration was demonstrated by testing the cell membrane permeability to fluorescent dyes [36]. Cell apoptosis induction was further illustrated by DNA fragmentation revealed with TUNEL staining [36] and by the activation of caspase-3 [40]. In our model, we also evidenced cell membrane permeabilization and caspase-3/7 activation using the ApoTox-Glo Triplex™ assay. Besides, we showed the absence of immediate cytotoxicity and thereby of necrosis measured by protease release except for the unspecific A2E toxicity above 45 μM (Fig. 3B). In ARPE-19 cells, apoptosis required A2E incubation at 100 µM followed by light stimulation while viability loss in human RPE-cells was achieved by sequential incubations with A2E-LDL complexes [38]. In our study, A2E incubation at concentrations higher than 45 μM were toxic for porcine RPE cells with an IC50 of 67 μM even in darkness. Moreover, light shifted the A2E dose-dependent cell apoptosis induction toward lower A2E concentrations.

Light-induced degeneration of A2E-loaded human RPE cells was reported under a broadband illumination from 390 to 550 nm with an irradiance of 2.8 mW/cm^2^ [38]. In this case, significant light toxicity was obtained after at least 72 hour light exposure. A similar broadband illumination from 390 to 750 nm but at a 2-log unit higher intensity (246 mW/cm^2^) was also toxic onto A2E-loaded ARPE-19 cells but within only 20 minute light exposure [35]. Furthermore, a restricted 15–60 second blue light exposure at 480 nm±20 nm onto ARPE-19 cells was found significantly more toxic than green light exposure at 545 nm±15 nm, even though cells were exposed to significantly higher irradiances with green light (210 mW/mm^2^) than with blue light (75 mW/mm^2^) [36]. As a consequence, the light-induced cell loss was greatly reduced (78 to 82% reduction) by attenuating blue light with an intraocular lens, which completely blocks light below 400 nm to linearly increase its transmittance up to 450 nm and then display a more shallow transmittance increase at higher wavelengths up to 800 nm [35]. More recently, cell protection was also demonstrated by filtering a spectral range from 390 to 460 nm [39]. Although all these studies clearly evidenced the greater toxicity of blue light, it did not enable investigators to define the most harmful wavelengths reaching the retina in averaged daylight. To answer this specific question, we have first calculated averaged sunlight irradiances reaching the retina by taking into account the eye media transmittance. Second, we have normalized irradiance levels for cell exposure with respect to the sunlight retinal irradiances. Practically, cells were exposed for 18 hours to 10 nm illumination bands (14 bands equally distributed within the blue-green range in 10 nm increments with the first band centered at 390 nm and going up to 520 nm). We thus demonstrated a cell viability loss at all tested wavelengths but with greater and more statistically significant differences under four 10 nm illumination bands centered at 420, 430, 440 and 450 nm. Cell apoptosis was also significantly increased under the same four illumination bands at the two highest A2E concentrations (20 µM and 40 µM). These results suggest that the 415–455 nm spectral range may be the most damaging light for patients at risk for ARMD. These results indicate that neither the wavelength energy nor the light intensity is the predominant factor in RPE cell photodamage. Instead, the wavelength-dependent phototoxicity is found to overlap with the visible absorbance maximum of A2E-loaded RPE cells. Therefore, blue light toxicity on RPE cells is likely a complex integration between light intensity, wavelength energy and the A2E absorption spectrum.

Light damages have been investigated by other research teams not only *in vitro* as discussed above but also *in vivo* in different animal models. For instance, they were examined under very intense light during a short period such as 3000 lux for up to 2 hours in albino rats. Photoreceptor degeneration occurred within less than 90 minutes whereas RPE cell apoptosis was delayed by several hours [44]. The use of two different animal models rhodopsin and RPE65 deficient mice indicated that rhodopsin activation is required to trigger this photoreceptor degeneration [45]. When different illumination bands were tested, blue light (403±10 nm) generated severe retinal damages whereas green light (550±10 nm) had no toxic effect [25]. This toxic effect of blue light was attributed to the photoreversal of rhodopsin bleaching, which can occur under blue but not green light increasing thereby the photon catch capacity of the retina [25]. This blue light damage of photoreceptor may therefore differ from the blue light damage of RPE cells. This conclusion was further supported by the observation of RPE cell death in blue light-exposed mice lacking rhodopsin [25]. However, this *in vivo* blue light damage of RPE cells could also differ from A2E-induced RPE cell damages because rhodopsin knockout mouse retina are unlikely to generate A2E formation as following RPE65 blockade [46]. This *in vivo* blue light damage of RPE cells could be more relevant to the RPE damage observed in our hands in the absence of A2E. However, the A2E-induced light damage of RPE cells could be relevant to the early RPE/photoreceptor dystrophy associated with light-induced photoreceptor degeneration recently described in mouse models with abnormal A2E accumulation [47], [48]. In fact, these mice were even considered animal models of ARMD as they display several major ARMD features such as lipofuscin accumulation, drusens, RPE cell death, complement activation and even choroidal neovascularization [49]. Future studies should therefore investigate if the phototoxic action spectrum defined in our *in vitro* study on A2E-loaded RPE cells is similarly more toxic during the light-induced degeneration in these ARMD animal models and whether filtering-out the corresponding wavelengths would efficiently prevent the RPE/photoreceptor degeneration and even suppress other complications.

Light involvement in the development of ARMD was suggested by epidemiological studies and by the beneficial influence of macular pigments as well as other antioxidants. Macular pigments are natural protective filters attenuating blue light in the range from 400 to 500 nm with peaks at 452 and 463 nm for lutein and zeaxanthin, respectively. Their absorptions and thus, their retinal concentrations, are known to decrease with age [50], [51], [52]. So, their high level dietary supplementation might diminish the risk of late ARMD [53] and even improve visual functions in severe ARMD patients [54]. All these conclusions are consistent with a mechanism of light-induced toxicity in the blue light range. In fact, this natural protection has been used to generate broad blue blocking filters and intraocular lenses [55], [56], [57], [58]. However, if the ARMD physiopathology relies on A2E photosensitization, our result suggests that filtering light in a narrower band from 415 nm to 455 nm may be sufficient to prevent or limit the disease development or progression. This more precise and narrower phototoxic action spectrum could be advantageously valued in selective photoprotection ophthalmic filters which would limit the disruption of color vision and of non-visual functions, by contrast to current blue filtering intraocular lenses [59]. Indeed, filters in our narrow bandwidth would not occlude light in the 460–500 nm range, not only essential for color vision but also for pupil constriction and circadian rhythm regulation, both mediated by melanopsin-sensitive retinal ganglion cells. Future studies will therefore have to evaluate if new selective ophthalmic filters in the here defined bandwidth from 415 nm to 455 nm could provide macular protection in patients at risk for ARMD.

## Materials and Methods

### Calculation of light conditions

To mimic physiological light conditions on the retina, RPE cells were exposed to a normalized light spectrum obtained by applying the ocular media filtering onto a referenced solar spectrum. The terrestrial standard reference solar spectrum ASTM G173-03 (International standard ISO 9845–1, 1992) was used, including the direct beam from the sun plus the circumsolar component in a 2.5° disk around the sun. The spectrum was deviated from the Simple Model of the Atmospheric Radiative Transfer of Sunshine (SMARTS) version 2.9.2. The retinal irradiance levels *E*_e_,_retina_ (W/m^2^) produced by sun exposure was determined using a simplified eye/light source model adapted from [42] (Fig. 1A). The light source is described by its energetic radiance *L*_e,λ,source_(λ) (W/sr/m^2^) measured in the pupil direction and its emitting surface *S*_source_ (m^2^). The source is assumed to be small compared to the distance u (m) between the source and the cornea. The cornea plane, the pupil plane and the nodal planes are assumed to be superimposed. The retina surface *S*_retina_ illuminated by the light source is proportional to the surface of the source *S*_source_, according to the formula:



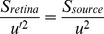



where u' is the length of the eye (m).

First, for each wavelength, the corneal energetic irradiance *E*_e,cornea_ (W/m^2^) produced by the emitting surface *S*_source_ of energetic radiance *L*_e,λ,source_ was calculated from the formula:



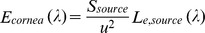



Next, for each wavelength, the radiant power entering the pupil was calculated from the formula:



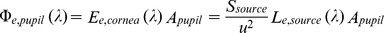



where *A*_pupil_ is the pupil surface (m^2^) and is calculated for a 5 mm pupil size.

Before reaching the retina, the radiant power is attenuated by the transmittance τ(λ) of the ocular media: cornea, aqueous humour, crystalline lens and vitreous (Fig. 1B). Thus, for each wavelength, the retinal irradiance level *E*_e_,_retina_(λ) was deduced of the radiant power entering the pupil *Φ*_e_,_pupil_(λ) by applying the natural filtering of the ocular tissues of a child [42], [60] (Fig. 1B–C):



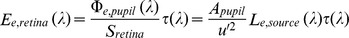



with a 5 mm pupil size and a 17 mm length eye:



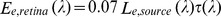



To accelerate light damage in our *in vitro* model, RPE cells were exposed for 18 hours to an irradiance 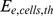 normalized to the calculated irradiances reaching the retina *E*_e_,_retina_ but multiplied by a factor f of 66 (Fig. 1D).
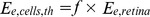



### Light emitting device

A specific LED-based fibered illumination device was especially designed to illuminate the RPE cells (Fig. 2A). A first optical unit (Unit 1, Fig. 2A) generated light under computer control to ensure gradient irradiance levels from 0 mW/cm^2^ up to 10 mW/cm^2^. Fifteen light channels were used, each composed of LEDs filtered by 10 nm bandwidth interferential band-pass filters. Fourteen bands were equally distributed in 10 nm increments within the blue-green range with the first band centered at 390 nm and going up to 520 nm. An additional band was set at 630 nm as a control.

The light generator was kept outside the incubator in order to prevent RPE cell growth disruption by heat production or vibrations. Five distinct illumination bands were generated and transmitted simultaneously by optical fiber bundles entering into the incubator onto the bottom of five subdivisions of the culture 96-well plate (Unit 2, Fig. 2A and B). Tapered light pipe homogenizing rods were used to provide uniform lighting condition on each subdivision composed of 16 wells (Fig. 2B). For each illumination channel, the software monitoring the irradiance levels was calibrated on the calculated irradiances reaching the cells 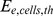 as described above. For each well plate, a 16-well subdivision was continuously maintained in darkness.

Irradiance level and uniformity measurements were successively assessed using (i) the calibrated spectroradiometer JAZ (Ocean Optics®, Dunedin, Fl, USA), with a cosine corrector probe and calibrated in Absolute Irradiance and (ii) an optical power meter (Coherent Inc®, Santa Clara, USA), with a photodiode with a total active area of 0.4 cm^2^. The quadratic derivative control included (i) the spectroradiometer precision, (ii) the well position on the subdivision and (iii) the repeatability study. The measured cell irradiances were consistent with the targeted ones. The 10 nm bandwidth spectral ranges were assessed using the spectroradiometer JAZ (Ocean Optics®) at least 4 and up to 6 times. Total irradiance was assessed before and after every experiment using the spectroradiometer JAZ (Ocean Optics®).

### A2E synthesis

A2E was synthesized by Orga-link (Magny-Les-Hameaux, France) using a modified procedure based on a previously described method [20]. Briefly, all-trans-retinal (880 μM–1 eq.), ethanolamine (387 μM–0.44 eq.) and acetic acid (387 μM–0.44 eq.) were mixed in absolute ethanol (8 ml) in darkness. The dark orange solution was stirred at room temperature during 7 days. The solvent was evaporated and the crude product (250 mg) was purified by preparative HPLC in the dark to isolate A2E (25.3 μM–5.8%) with a purity of 98% HPLC. A2E was stored at −80°C in dark vials after dilution in DMSO at 50 mM final concentration.

### Light exposure of A2E-loaded RPE cells

Porcine eyes were bought at a local slaughterhouse (Guy Harang, Houdan, France) in agreement with the local regulatory department and the slaughterhouse veterinarians. This procedure adheres to the european initiative for restricting animal experimentation because not a single animal was killed for our experimentation. Eyes were taken from animals daily sacrificed for human consumption. Eyes were cleaned from muscle, and incubated during 4 minutes in Pursept-AXpress (Merz Hygiene GmbH, Frankfurt, Germany) for disinfection. The anterior portion was cut along the limbus to remove the cornea, lens and retina. A solution containing 0.25% trypsin-EDTA (Life Technologies, Carlsbad, CA, USA) was introduced for 1 hour at 37°C in the eyecup. RPE cells were then gently detached from the Bruch's membrane and resuspended in Dulbecco's Modified Eagle medium (DMEM, Life Technologies) supplemented with 20% Fetal Bovine Serum (FBS, Life Technologies) and 10 µg/ml gentamycin (Life Technologies). Purified cells from 6 to 8 eyes were pooled and plated in as many 60 mm Petri dishes as prepared eyes. Cells were allowed to grow in an incubator with a controlled atmosphere at 5% CO2 and 37°C. The culture medium was renewed 24 hours after the first seeding. When cells reached confluence (about 3 days), they were detached from the Petri dish by a 5 minute 0.05% trypsin-EDTA treatment at 37°C. They were then resuspended in DMEM supplemented with 20% FBS and 10 µg/ml gentamycin and seeded in a black clear bottom 96-well plate at 75,000 cells per well.

A2E was added to the culture medium 72 hours after the seeding in 96-well plate when RPE cells had become confluent. The DMEM-20% SVF medium was removed and exchanged for DMEM without serum but containing A2E from 0 to 100 µM. In all conditions, the final DMSO concentration was adjusted to 0.1%. Six hours later, cells were washed twice in modified DMEM without any photosensitizer such as phenol red, riboflavin, folic acid and aromatic amino acids.

The dose-response curve of A2E toxicity was assessed with the CellTiter-Glo® Luminescent Cell Viability Assay (Promega, Fitchburg, WI, USA) according to the manufacturer protocol. Subsequently, cell viability was quantified by the ApoTox-Glo™ assay (Promega), which provides also information on apoptosis and necrosis. For phototoxic action spectrum experiments, cells were exposed to light for 18 hours and then kept in darkness for 6 hours prior to the ApoTox-Glo™ assay (Promega). All measures were averaged from 4 wells for each illumination band and each A2E concentration. The values were then normalized to the control value in darkness. Measurements performed on an Infinite® M1000 microplate reader (TECAN, Männedorf, Switzerland) were reiterated for each illumination band in 4 to 6 independent experiments.

### Quantification of A2E in RPE cells

Cells were incubated for 6 hours in 5, 15, 20, 30 or 40 µM of A2E. Then, they were washed 3 times with modified DMEM to remove the remaining extracellular A2E. Cells were then retrieved by a 7 minute 0.05% trypsin-EDTA treatment and resuspended in phosphate-buffered saline solution (PBS, Life Technologies). Cells were mixed twice with a Polytron PT MR2100 (Kinematica AG, Luzern, Switzerland) during 45 seconds and kept at −80°C in dark vials. All these steps were carried out in the dark under red light.

Unfrozen RPE cell extracts were homogenized with a solution of chloroform-methanol (2:1 ml). A2E was extracted three times using the same organic solution. After removing all solvent in vacuum, each sample was dissolved in methanol (200 μl) and filtered (0.2 μm) before UPLC analysis. For UPLC analysis, standards and samples were injected onto a reversed phase (C18) column (HSS C18, Waters, 2.1×50 mm–1.7 μm), and eluted with a gradient of methanol in water (80–92% methanol +0.1% HCOOH, 0.6 ml/min, Waters UPLC Acquity system). A triple quadrupole mass spectroscopy detector (Waters) on SIR Mode (Single Ion Recording – m/z = 592.45– M+) was used for A2E quantification. The A2E concentration in RPE cells was determined from integrated peak areas and expressed as nanograms per 10^5^ cells. The A2E intracellular quantification was repeated in 4 independent experiments.

### Autofluorescence imaging of RPE cells

A2E autofluorescence imaging was performed on fixed cells after A2E incubation at various concentrations from 0 to 100 µM in Lab-Tek 8-chambers slide (Thermo Fisher Scientific, Waltham, MA, USA). Cells were fixed 15 minutes at 4°C with 4% (wt/vol) paraformaldehyde in PBS (0.01 M, pH 7.4). Cell nuclei were revealed incubating cells with 10 µg/ml 4′, 6-Diamidino-2-phenylindole (DAPI, Sigma-Aldrich, Saint-Louis, Mo, USA) for 1 hour at room temperature. Then cells were rinsed and mounted with Permafluor® reagent (Microm, Francheville, France). Images were acquired with an Olympus FV1000 upright confocal microscope using identical settings for each A2E concentration and a 40× objective with 488/520 nm excitation/emission filters. Images of DAPI stained nuclei were obtained using 405/461 nm excitation/emission filters.

### Absorbance spectra of A2E

Absorbance spectra of A2E were measured with the TECAN infinite M1000 microplate reader, either on free dissolved A2E, or on RPE cells. Free A2E was dissolved in ethanol or in modified DMEM. The absorbance spectrum was measured on living RPE cells incubated for 6 hours in modified DMEM containing 40 µM A2E and then washed 3 times.

### Statistical analysis

Statistical analysis of light effect on RPE cell viability, caspase-3/7 activities and necrosis were performed using Graph Pad Prism 6 software. When precised, statistical analysis was performed using t-test. Otherwise, one-way ANOVA was used to compare variances between groups at each A2E concentration. In case of significant differences, the means for each wavelength was compared to the dark control group with a Dunnett multiple comparison test. Differences were considered significant at *p<0.05, **p<0.01 and ***p<0.001.
